# Progress in Medicine

**Published:** 1899-05-06

**Authors:** 


### Progress in Medicine.

### DISEASES OF THE NERVOUS SYSTEM.

(*Continued from page* 64.)

**Head-nodding in Infants.**—Aldrich[6] has recently reported two cases of this curious affection and given a useful *résumé* of our knowledge of the condition, to which but little has been added since the publication of important papers on the subject by the late Dr. Hadden in 1890. The disorder occurs in the majority of cases between the ages of six and twelve months, though isolated ones as early as six weeks and as late as three years have been recorded. The movements of the head are in rare cases those of assent, like the nodding of a mandarin doll; as a rule these are of dissent, *i.e.*, the head is rotated round a vertical axis to and fro. These movements are singularly smooth, easy, regular, and rhythmical, and free from any spasmodic feature; they stop during sleep, in the recumbent posture, and in some cases at least when the eyes are covered. They stop for a few seconds if the attention is strongly roused. These gyral movements of the head are almost constantly associated with nystagmus, which may be monocular or binocular, vertical, rotary, or mixed and alternating. Binocular nystagmus is the most common. In purely gyral movements of the head we are more apt to have a horizontal nystagmus, whilst in pure cases of head-nodding (movements of assent) the nystagmus is usually vertical. The nystagmus is rendered more marked or, if absent, sometimes brought out by fixing the head; it is not modified or inhibited by change of posture, in this respect differing from the head movements. The nystagmus usually precedes the development of the head movements, often persists long after the latter have disappeared, and may recur without their association. A few of the cases have had a squint. The optic discs are normal, and vision, as far as one can tell, is not necessarily at fault. Some of the patients have had a curious way of looking at objects, *e.g.*, one turned its head away and looked sideways. No epileptic fits have been observed in any of the cases, and, unlike epilepsy, no case has been recorded that did not recover. The exciting causes of this curious disorder are but little known; dentition is very doubtful, for many cases begin long before this begins; rickets and falls on the head are probably not more common or marked in these cases than in those infants who do not suffer from the disorder. The pathology of such a functional disease as this, which possesses an inherent tendency to self-cure, rests on a basis of analogy rather than on histological fact. Hadden thought that the disorder is due to instability of motor centres above the nuclei in the spinal cord and fourth ventricle, *i.e.*, to functional disturbance in the cerebral cortex. The child acquires certain voluntary movements of the head and eyeballs, and the disturbance takes place during the process of this psychical development, which occurs coincidently with the development and complete medullation of the central and peripheral neurons and their mutual connection. The diagnosis is easy, the monotonously smooth nodding and gyral movements are quite unlike the spasmodic twitching of epilepsy and the purposive movements of habit spasm and chorea; and the discovery of the associated nystagmus settles the question. The prognosis as regards life is good, and most cases tend to spontaneous cure in from six weeks to three months, though many relapse if the child suffers any disturbance of health. The treatment which has seemed to expedite recovery is administration of bromides, and correction of any gastro-intestinal affection or associated rickets.

**Epilepsy.**—Hughlings Jackson[7] has recently put forward a new theory to account for the asyphxia of slight epileptic paroxysms. In severe fits the asphyxia may reasonably be considered due to the arrest of respiration from spasmodic fixation of the chest which takes place. In slight fits another explanation is possible. Spencer showed that stimulation of the cortex on the outer side of the olfactory tract, and just in front of the junction of this tract with the uncinate, produces an arrest of respiration nearly always in the expiratory position. This arrest is probably due to cortical inhibition of the medullary respiratory centre. Now, in many slight epileptic fits there is evidence that the discharge takes place from this portion of the cortex; thus, there may be a crude sensation of smell or taste, or there may be movements of chewing, smacking the lips, and sometimes spitting, which are indirect results of an epileptic discharge of gustatory elements of the cortex. And, according to Ferrier, the representation of smell and taste is in the uncinate gyrus. So that it seems very probable that in epileptic fits, where evidence of discharge of the cortical centres of taste and smell is forthcoming, the asphyxia is due to arrest of respiration from stimulation of the centre discovered by Spencer, and not to fixation of the chest.

**Auto-Intoxication in Epilepsy.**—Solaro[8] finds probable evidence of auto-intoxication in epilepsy in the aura and in the supervention of dementia in the dyspeptic disorders which often precedes an attack, such as vomiting, fœtor of breath, constipation, and diarrhœa. Again, the toxicity of the urine is less during an attack and greater afterwards, and the elimination of uric acid is less before and greater after. Treatment addressed to the eliminating organs, to the digestive tract, receives fresh sanction on the view that auto-intoxication plays no inconsiderable part in the production of the epileptic attack. The rôle of bromides in calming the cerebral activity is in no way lessened by laying stress on the importance of getting rid of toxic substances in the organism.

**Treatment of Epilepsy.**—Upson[9] considers bromide of strontium likely to supplant the other bromides in consequence of its being more easily tolerated and free from depressant effects. Amongst other drugs, he speaks highly of trional in 3 or 4 grain doses three or four times a day when bromides are ill borne. He has never seen its continued use followed by bad symptoms. It may be combined with bromide of strontium. Digitalis is a useful adjunct to other remedies when there is a low tension pulse. Belladonna is of limited service in milder cases. He has seen no good results from antipyrin or other coal tar products. Strychnine is to be avoided even as a small dose in laxative pills. Quinine should be avoided. Nitrite of amyl may cut short the attack when there is an appreciable warning; and the same, he thinks, may be said of the ligature or encircling blister, which may be used when the aura travels up the arm or leg. Keeping the bowels clear, great regularity in the mode of life, avoidance of fatigue and emotion, and the utmost care in alcoholic liquors are enjoined.

[6] Aldrich, Amer. Jour. Med. Sci., Feb. 1899. [7] Jackson, Lancet, Jan. 14, 1899. [8] Solaro, Rif. Med., July 19, 20, 21, 1898. [9] Upson, Cleveland Med. Gaz., Oct., 1898.

